# Parahippocampal Involvement in Mesial Temporal Lobe Epilepsy with Hippocampal Sclerosis: A Proof of Concept from Memory-Guided Saccades

**DOI:** 10.3389/fneur.2017.00595

**Published:** 2017-11-07

**Authors:** Silvia Colnaghi, Giorgio Beltrami, Guy Poloni, Anna Pichiecchio, Stefano Bastianello, Carlo Andrea Galimberti, Maurizio Versino

**Affiliations:** ^1^Laboratory of Neuro-otology and Neuro-ophtalmology, Fondazione Istituto Neurologico Nazionale Casimiro Mondino (IRCCS), Pavia, Italy; ^2^Department of Public Health, Experimental and Forensic Medicine, University of Pavia, Pavia, Italy; ^3^Department of Electrical, Computer and Biomedical Engineering, University of Pavia, Pavia, Italy; ^4^Neuroradiology Department, Fondazione Istituto Neurologico Nazionale Casimiro Mondino (IRCCS), Pavia, Italy; ^5^Department of Brain and Behavioral Sciences, University of Pavia, Pavia, Italy; ^6^Epilepsy Centre, Fondazione Istituto Neurologico Nazionale Casimiro Mondino (IRCCS), Pavia, Italy

**Keywords:** memory-guided saccade, voxel-based morphometry, spatial memory, parahippocampal cortex, mesial temporal lobe epilepsy

## Abstract

**Objective:**

Mesial temporal lobe epilepsy with hippocampal sclerosis (MTLE-HS) may involve extrahippocampal areas of structural damage and dysfunction. The accuracy of medium-term spatial memory can be tested by memory-guided saccades (MGS) to evaluate a functional impairment of the parahippocampal cortex (PHC), while voxel-based morphometry (VBM) analysis can be used to detect a structural damage of the latter region.

**Methods:**

MGS with 3- and 30-s memorization delays were compared between 7 patients affected by right MTLE-HS (r-MTLE-HS), 6 patients affected by left MTLE-HS, and 13 healthy controls. The same subjects underwent brain MRI for a VBM analysis. Correlation analysis was performed between the results of VBM and MGS and with patients’ clinical data.

**Results:**

Right MTLE-HS patients showed impaired accuracy of leftward MGS with a 30-s memorization delay; their gray-matter volume was reduced in the right hippocampus and inferior temporal gyrus, and bilaterally in the cerebellum. Left MTLE-HS patients had normal MGS accuracy; their gray-matter volume was reduced in the left hippocampus, in the right-inferior temporal gyrus and corpus callosus, and bilaterally in the insular cortex and in the cerebellum. The difference between right and left parahippocampal volumes correlated with MGS accuracy, while right and left hippocampal volumes did not. Hippocampal and parahippocampal volume did not correlate with clinical variables such as febrile seizures, age at disease onset, disease duration, and seizure frequency.

**Conclusion:**

MGS abnormalities suggested the functional involvement of the right PHC in patients with r-MTLE-HS, supporting a right lateralization of spatial memory control and showing a relation between functional impairment and degree of atrophy.

## Introduction

Extrahippocampal areas of structural damage may be detected in mesial temporal lobe epilepsy with hippocampal sclerosis (MTLE-HS), including the parahippocampal and mesial temporal lobe cortex (1–5).

These findings have been emphasized in the MTLE-HS workshop promoted by the International League Against Epilepsy (6), when the importance of defining the site and characteristics of extrahippocampal damage was underlined. In fact, structural damage of these areas has an incidence that varies in relation with the different diagnostic methods, and the characteristics of extrahippocampal pathology in MTLE-HS (6) as well as its pathogenesis are still a matter of debate.

Here, we aimed at evaluating the consistency of neurophysiological data indicating a functional involvement of the parahippocampal cortex (PHC) with voxel-based morphometry (VBM) data.

Many brain regions have been shown to be reduced in volume in temporal lobe epilepsy (TLE) patients with respect to healthy subjects [see Ref. (7) for a review]. Temporal lobe abnormalities were mainly ipsilateral to the epileptic focus, while extratemporal and subcortical abnormalities were bilateral. This distribution of brain abnormalities in TLE patients is consistent with postmortem and fMRI imaging results. Hippocampal atrophy ipsilateral to the epileptic focus is the most common neuropathological correlate of TLE (8), and patients with right-sided epileptic focus are more likely to have bilateral hippocampal volume reduction (9).

A recent study (10) showed that history of febrile convulsions (FC), dystonic posturing, and secondary generalized tonic–clonic seizures are cardinal criteria that could be reliably helpful to distinguish TLE patients with hippocampal sclerosis from those with other TLE (i.e., patients with mesial structural lesion other than hippocampal sclerosis and MRI-negative cases), suggesting that MTLE-HS could be considered as a distinctive syndrome. When HS is detectable, patients with MTLE showed an earlier epilepsy onset, exhibited more frequently early febrile seizures (FS), and presented more ictal gestural automatisms, dystonic posturing and secondary generalized tonic–clonic seizures.

Mesial temporal lobe epilepsy with hippocampal sclerosis patients show material-specific memory impairment depending on their hemispheric language dominance. For instance, verbal memory impairment was found in left MTLE-HS (l-MTLE-HS) patients with left language dominant hemisphere, while a weaker association was found between visual memory impairment and right temporal dysfunction (11, 12) in right MTLE-HS (r-MTLE-HS) patients.

The memory-guided saccades (MGS) can be used to study cortical control of short and medium-term spatial memory in humans (13, 14). In the MGS paradigm, subjects are requested to make a volitional saccade directed toward a location in which a target was previously present.

Functional imagery, transcranial magnetic stimulation, and lesion studies have been used to obtain a spatially and temporally accurate model of the MGS cortical control in normal subjects and in patients with lesions of the temporal lobe structures (15).

In particular, it has been showed that accuracy of MGS with memorization delays from 1 to 20 s depends on the dorsolateral prefrontal cortex (DLPFC) (16), while the PHC is responsible for the accuracy of MGS with memorization delays longer than 20 s and up to a few minutes (16–19). Ploner et al. (18) exploited the MGS paradigm to test the role of the PHC for the accuracy of spatial memory in humans. They recorded the MGS with delays op to 30 s in patients that underwent resection of the right mesial temporal lobe for intractable epilepsy. Patients whose lesion was limited to the PHC made amplitude error of memory guided eye movements with 30-s delay (30 MGS) directed contralateral to the lesion side, while patient as whose resection included the perirhinal cortex but not the PHC were able to perform the MGS with no such errors. Taking into account these findings, in a previous study (20) we recorded the MGS with memorization delays of 3 (3 MGS) and 30 s in patients with r-MTLE-HS and we found a delay-dependent inaccuracy of 30 MGS contralateral to the lesion suggesting a functional impairment of the right PHC.

Here, we hypothesized that the accuracy of 30 MGS directed contralaterally to the epilepsy focus could be impaired in MTLE-HS patients with VBM signs of structural involvement of extrahippocampal brain regions, particularly of the PHC of the ipsilateral mesial temporal lobe, and that this impairment could be associated with clinical data, particularly disease duration, and seizure frequency.

## Materials and Methods

### Subjects

Thirteen right-handed subjects with r-MTLE-HS (*n* = 7) or l-MTLE-HS (*n* = 6) and 13 healthy subjects underwent recording of 3 MGS and 30 MGS and brain MRI for VBM quantitative analysis.

Mesial temporal lobe epilepsy with hippocampal sclerosis patients were recruited among the outpatients consecutively referred to the Epilepsy Centre of the Neurological Institute C. Mondino of Pavia.

We excluded patients unable to participate due to difficulties in understanding the experimental procedures or in maintaining attention for a long time, those older than 60 years, those with a seizure frequency more than 2 per week, and those who modified antiepileptic treatment in the previous month or experienced an epileptic seizure in the previous 36 h. Exclusion criteria were chosen in order to avoid gray mater reduction associated with age (21) and biases in eye movement test performance possibly due to post-ictal dysfunction.

Patients’ demographical and clinical data are shown in Table 1.

**Table 1:** Demographic and clinical features of patients with right (r-MTLE-HS) and left (l-MTLE-HS) mesial temporal lobe epilepsy with hippocampal sclerosis.

	Age (decade)	Age at epilepsy onset (years or months)	Disease duration (years)	FS	AED	Seizure frequency (per month)
r-MTLE-HS	6th	43 years	9	y	PHT 325 LEV 4000 CLB 20	1.5

	4th	7 months	20	n	CBZ CR 800 PHT 200 CLB 30	4.5

	6th	37 years	15	y	LTG 600 CLB 20 LEV 500	1.5

	4th	9 month	38	y	CBZ CR 1100 LTG 500	8.5

	4th	29 years	7	y	CBZ CR 800 LTG 200	3

	7th	6 month	35	n	CBZ CR 1000 LEV 3500 PHT 0.5	3

	3th	14 years	15	n	LTG 550 LEV 3000	2.5

l-MTLE-HS	5th	23 years	23	n	CLB 10 OXC 600	0.1

	5th	14 years	28	y	LTG 500 CBZ 900 LEV 550	2.5

	4th	8 years	22	y	CBZ 800 LEV 3000	0.1

	6th	20 years	34	n	CBZ 800	0.1

	6th	22 years	36	y	CBZ 800 LEV 3000	0.25

	4th	15 years	22	n	LTG 600 CBZ 400 LEV 3000	4.50

*M, male; F, female; FS, febrile seizures; n, no; y, yes. AED, antiepileptic drug treatment, mg daily; CBZ, carbamazepine; LTG, lamotrigine; LEV, levetiracetam; OXC, oxcarbazepine; PHT, fenitoine; CLB, clobazam. Seizure frequency = monthly mean calculated on the basis of the seizure reported in the last year on the patient’s seizure diary*.

Epilepsy diagnosis was supported by clinical, electroencephalography (EEG), and MRI criteria. More in detail, conventional brain MRI showed atrophy and T2 signal increase that were limited to the hippocampal formation in each patient, and did not show parahippocampal atrophy in any of them. EEG traces, ictal symptoms, and signs suggested a mesial temporal lobe seizure onset in each patient (6).

All patients were on treatment with antiepileptic drugs with serum levels in therapeutic range.

Thirteen right-handed healthy subjects (seven women and six men; mean age: 38.4 years, SD: 10.5, range: 27–60 years) age-matched with the patient group (mean age: 43.8 years, SD 11.0, range: 29–60 years) were included in the control group.

The research protocol received approval from the local Ethics Committee and all the procedures were conducted in accordance with the Declaration of Helsinki. All the subjects gave written informed consent before participating in the study.

### Memory-Guided Saccades

The eye movements were calibrated and recorded monocularly from the right eye with the scleral search coil technique (22) (SKALAR system S3020: spatial resolution better than 0.1°; sampling rate 250 Hz, bandwidth 0–70 Hz).

The eye movement recording sessions, data acquisition, and analysis were the same as reported in our previous study (20).

The subjects were seated in a dark room with their head in the upright position on a chinrest. For every subject, we recorded the reflexive saccades (RS), the 3 MGS, and the 30 MGS in three separate sessions. In each session, every subject performed 18 trials in both directions (leftward and rightward saccades) for a total of 108 trials each.

In the RS paradigm, a horizontally presented lateral target with an unpredictable direction and amplitude (10°, 15°, or 20°) was lit for 2 s, while the subject was staring at a central point. The subjects were instructed to look at this light immediately after its appearance and until it disappeared. The next trial began at the central fixation point.

In the MGS paradigm, the subjects tried to memorize the location of a horizontally presented lateral target lit for 200 ms, while they were staring at the central point. The target had unpredictable direction and amplitude (10°, 15°, or 20°). After the memorization delay of 3 or 30 s, the central fixation point was switched off, which was the signal for the subject to perform a saccade toward the memorized location. The previously flashed target was shown again after 2 s and the subject had to make a corrective saccade if necessary. The next trial began at the central fixation point.

Memory-guided saccades trials with prosaccades, namely erroneous RS directed at the flashed target, were excluded from analysis. We used a custom-made program developed with LabView software (National Instruments, Austin, TX, USA) to analyze the saccades offline by identifying the beginning and the end of each saccade based on threshold velocity criteria; the difference in the eye position at these two points corresponded to the pulse amplitude. The operator positioned one additional mark that identified the final position, namely the position the eye reached after all the corrective saccades and before the reappearance of the target; the difference between the starting and final positions corresponded to the final amplitude.

We computed the following equations:

1. the saccade accuracies (SA) as
  $$\begin{array}{c} \text{pulseSA }=\text{ pulse amplitude}/\text{target amplitude},\\ \text{finalSA }=\text{ final amplitude}/\text{target amplitude} \end{array}$$
2. the amplitude errors (E) as
  $$\begin{array}{c} \text{pulseE }=\text{ ln}|\text{1}-\text{ pulseSA}|\text{,}\\ \text{finalE}=\text{ln}|1-\text{ finalSA}| \end{array}$$
3. the amplitude error difference (ED) as

ED=finalE−pulseE.

A logarithmic transformation was needed in order to approximate a normal distribution of the values, and we used the absolute value of the ǀ 1 − SA ǀ differences to express a scatter of the MGS endpoints despite the presence of both hypometric and hypermetric saccades.

For each subject, paradigm, and saccade direction, we computed the mean value of latency, SA, *E*, and ED.

The patients were compared with the controls as a group by using repeated measure analyses of variance on all the parameters listed before. The RS and the 3- and 30-s MGS were analyzed separately. The analyses considered one intra-individual factor (saccade direction: right or left), one inter-individual factor (group: controls or patients), and their interactions.

The significance value was set at *p* = 0.05.

We used chi-square test to evaluate the occurrence of patients whose mean value exceeded the normal range, which was calculated on the control group as the mean ± 2.5 SDs.

### Voxel-Based Morphometry

All the subjects’ MRI were performed with the same machine (Philips Intera Gyroscan 1.5 T), using coronal T1 Gradient Echo sequences, with identical acquisition parameters [echo time (TE) 4.6 ms, repetition time (TR) = 25 ms, thickness = 1.6 mm, slice gap = 0.8 mm, matrix = 256 × 256, voxel = 0.9 mm × 0.9 mm × 0.8 mm, means = 1].

The VBM study was performed through a between-groups comparison by dividing the subjects in three groups on the basis of presence and side of the hippocampal atrophy: controls (Ctrls), r-MTLE-HS (seven subjects), and l-MTLE-HS (six subjects). The areas of reduced gray-matter volume were identified first, then a study on the hippocampal region was performed: the mean volume of the hippocampal gray matter on each side and the difference in volume between the two sides were correlated with the clinical data (age at epilepsy onset, epilepsy duration, seizure frequency, antiepileptic drugs) and the results of MGS recording.

Neuroimage processing was done by statistical parametric map (SPM) (discrete cosine transform cutoff 8 mm) and MATLAB 7.4 programs. Acquired images were normalized on a whole-brain standard template MNI 152, with masking of the hippocampal region (7). Normalized images were segmented through the standard gray and white matter (GM/WM) templates from SPM. Filter value was set at 6-mm smoothing kernel, as suggested for studying structures with dimensions comparable with the hippocampus (7).

Normalized, segmented, and smoothed images were weighted regarding confounding variables (sex, age, and total brain volume). After the described preprocessing and normalization operations, anatomical images underwent a voxel-wise statistical analysis aimed at identifying differences between the three groups.

Statistical parametric maps of the whole brain were created for several comparisons: Ctrls vs. r-MTLE-HS, Ctrls vs. l-MTLE-HS, and l-MTLE-HS vs. r-MTLE-HS. All contrasted images were created using a *p* < 0.005.

We correlated the hippocampal and parahippocampal gray-matter volumes with the clinical and MGS parameters, and compared hippocampal and parahippocampal volumes of MTLE-HS patients with and without abnormal MGS.

## Results

### Saccades

Accuracy parameters’ mean and SE values are shown in Table 2.

**Table 2:** Mean and SE values of rightward and leftward saccades pulse and final amplitude, natural logarithm of pulse and finalError, and error difference (ED) between final and pulse for each diagnostic group (healthy controls = Ctrls, patients with right temporal lobe epilepsy and hyoppocampal sclerosis = r-MTLE-HS, and patients with left temporal lobe epilepsy and hyoppocampal sclerosis = l-MTLE-HS) in reflexive saccades (RS), 3 s (3 MGS) and 30 s (30 MGS) memory-guided saccades.

Side	Paradigm	Group	Amplitude				Error				ED	
			Pulse		Final		Pulse		Final		Final-Pulse	
			Mean	SE	Mean	SE	Mean	SE	Mean	SE	Mean	SE
RIGHTWARD	RS	Ctrls	0.907	0.019	0.986	0.011	−2.659	0.196	−4.178	0.201	−1.519	0.177
		r-MTLE-HS	0.909	0.020	0.970	0.009	−2.346	0.165	−3.738	0.369	−1.392	0.237
		l-MTLE-HS	0.843	0.021	0.959	0.016	−2.230	0.207	−3.858	0.277	−1.628	0.204

	3 MGS	Ctrls	0.868	0.033	0.944	0.021	−2.059	0.127	−2.579	0.095	−0.520	0.137
		r-MTLE-HS	0.899	0.058	0.962	0.019	−1.875	0.170	−2.438	0.237	−0.564	0.201
		l-MTLE-HS	0.845	0.024	0.996	0.046	−2.002	0.196	−2.722	0.231	−0.721	0.321

	30 MGS	Ctrls	0.778	0.049	0.940	0.029	−1.606	0.141	−2.363	0.180	−0.756	0.121
		r-MTLE-HS	0.875	0.097	0.889	0.085	−1.610	0.236	−1.852	0.120	−0.242	0.196
		l-MTLE-HS	0.827	0.103	0.928	0.095	−1.578	0.282	−2.121	0.252	−0.543	0.266

LEFTWARD	RS	Ctrls	0.878	0.016	0.970	0.000	−2.318	0.140	−3.738	0.200	−1.420	0.163
		r-MTLE-HS	0.874	0.008	0.953	0.000	−2.180	0.000	−3.229	0.000	−1.049	0.201
		l-MTLE-HS	0.836	0.032	0.949	0.000	−2.036	0.000	−3.363	0.000	−1.328	0.158

	3 MGS	Ctrls	0.833	0.033	0.921	0.024	−1.875	0.140	−2.652	0.193	−0.777	0.156
		r-MTLE-HS	0.851	0.037	0.987	0.044	−1.835	0.201	−2.604	0.195	−0.769	0.174
		l-MTLE-HS	0.879	0.047	0.988	0.040	−1.813	0.257	−2.491	0.155	−0.678	0.244

	30 MGS	Ctrls	0.826	0.056	0.919	0.026	−1.644	0.141	−2.465	0.174	−0.821	0.131
		r-MTLE-HS	0.875	0.059	0.980	0.050	−2.092	0.338	−2.151	0.096	−0.059	0.310
		l-MTLE-HS	0.943	0.093	0.987	0.057	−1.886	0.186	−2.556	0.155	−0.671	0.207

The effects of saccade direction and experimental group on all saccadic parameters are shown in Table 3.

**Table 3:** Effects of saccade direction, diagnostic group, and direction*group interaction on saccade peak velocity, latency, pulseError, finalError, and pulseError–finalError difference in reflexive saccades (RS), and 3- and 30-s memory-guided saccades (3 MGS and 30 MGS). Bold font is used to highlight the statistically significant comparisons.

Saccade parameter	Saccade kind	Direction effect		Group effect		Direction*group interaction	
		F	p	F	p	F	p
Peak velocity	RS	**5.114**	**0.033**	0.440	0.648	2.310	0.122
	3 MGS	**8.301**	**0.008**	0.428	0.657	0.029	0.972
	30 MGS	**14.325**	**0.001**	0.937	0.406	1.169	0.329

Latency	RS	0.481	0.495	0.382	0.687	0.114	0.893
	3 MGS	1.162	0.292	0.133	0.876	0.873	0.431
	30 MGS	1.009	0.744	1.117	0.360	0.797	0.721

PulseE	RS	3.279	0.083	1.540	0.235	0.019	0.797
	3 MGS	1.554	0.225	0.208	0.814	0.341	0.715
	30 MGS	**5.691**	**0.025**	0.437	0.651	1.543	0.234

FinalE	RS	**8.456**	**0.008**	1.250	0.305	0.012	0.988
	3 MGS	**8.456**	**0.008**	0.130	0.878	0.702	0.505
	30 MGS	**7.133**	**0.013**	1.555	0.232	1.010	0.379

ED	RS	4.090	0.054	0.298	0.745	0.479	0.625
	3 MGS	0.884	0.357	0.030	0.971	0.379	0.688
	30 MGS	0.000	0.988	4.992	0.015	0.413	0.666

The peak velocity values were significantly influenced by saccade direction being larger for rightward than for leftward saccades for all kinds of saccades, with no significant effect of group or group*direction interaction.

The latency values of RS, 3 MGS, and 30 MGS were not significantly influenced by direction, group, or group*direction interaction.

The pulseE values of both RS and 3 MGS were not significantly influenced by saccade direction, group, or group*direction interaction, whereas the pulseE of 30 MGS was significantly influenced by saccade direction being larger for rightward than for leftward saccades, with no significant effect of group or group*direction interaction.

The finalE values showed a different behavior depending on the different kinds of saccades.

For RS it proved to be smaller to the right than to the left, showing a significant direction effect with no significant effect of group and direction*group interaction. The finalE values of 3 MGS were not significantly influenced by saccade direction, group, or group*direction interaction. The finalE values of 30 MGS showed the same behavior detectable for pulseE of the same kind of saccades, since they were significantly influenced by saccade direction being larger to the right than to the left, with no significant effect of group or group*direction interaction.

The ED values of both RS and 3 MGS were not significantly influenced by saccade direction, group, or group*direction interaction, whereas the ED of 30 MGS was significantly influenced by group. This effect was mainly attributed to r-MTLE-HS patients who were unable to reduce the pulseE, and hence to minimize ED, as effectively as controls for leftward saccades (Sheffè *post hoc* test: *p* = 0.035), but a similar trend was detectable in r-MTLE-HS patients for rightward saccades also.

Thereby, the results of group analyses can be summarized as follows:

– RS pulseE and ED values were independent from direction, group, and their interaction, while finalE values were smaller for rightward than for leftward saccades both in controls and in patients.
– 3 MGS pulseE, finale, and ED values were independent from direction, group, and their interaction.
– 30 MGS pulseE and finalE values were larger for rightward than for leftward saccades both in controls and in patients; moreover, patients with r-MTLE-HS showed a larger ED than controls.

**Figure 1:**
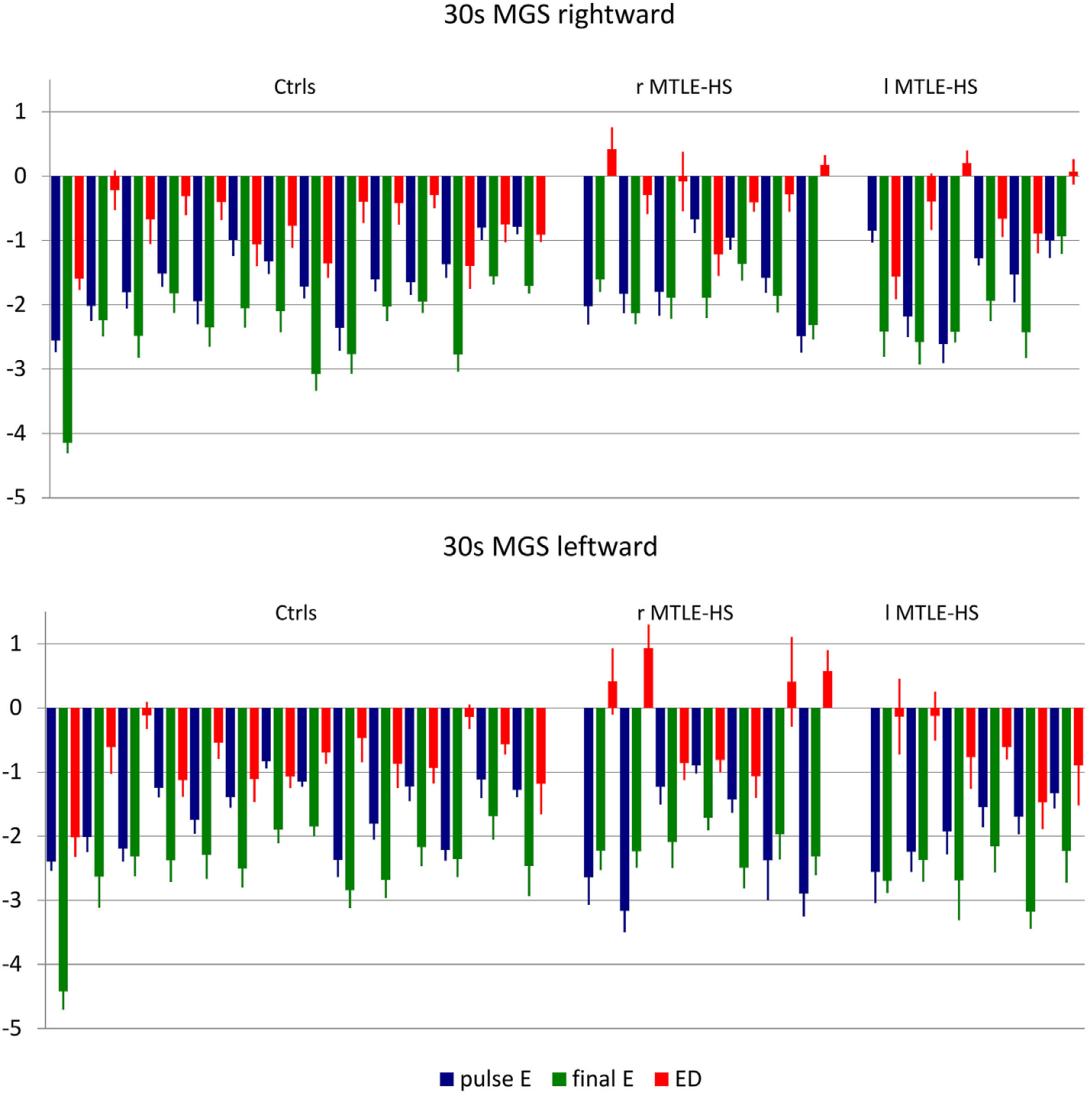
Mean and SE values of pulseError (pulseE), finalError (finalE), and error difference between pulse and final (ED) of 30-s delay memory-guided saccades (30 MGS) directed rightward (upper panel) and leftward (lower panel) for each subject in each diagnostic group. ctrls, healthy controls; r-MTLE-HS, patients with right-sided mesial temporal lobe epilepsy with hippocampal sclerosis; l-MTLE-HS, patients with left-sided mesial temporal lobe epilepsy with hippocampal sclerosis.

We also considered the patients individually; that is, we checked if accuracy values of their saccades fell within the normal limits defined as the mean ± 2.5 SDs computed in the control group (Figure 1).

The pulseE was invariably normal both in the control and in the patient groups for all kind of saccades.

The finalE was abnormal in a few subjects: two subjects (1 r-MTLE-HS and 1 control) for r-RS, 1 r-MTLE-HS patient for r-3MSG, no subjects for 30 MGS: the chi-square test showed that the distribution of abnormalities did not differ between patients and controls.

The ED was abnormal only in one l-MTLE-HS patient for r-3MGS: again, the chi-square test showed that the distribution of abnormalities did not differ in the patients as compared with controls.

The main finding derives from 30 MGS that showed an abnormal ED in four r-MTLE-HS patients (all of them for leftward saccades and in one of them for rightward saccades also) and in none of the l-MTLE patients or of the controls: the higher occurrence of abnormalities in the r-MTLE-HS group proved to be statistically significant (Fisher’ exact test *p* = 0.003).

### Voxel-Based Morphometry

Table 4 displays the results of gray-matter changes.

**Table 4:** Regions of statistically significant difference between groups.

Relative atrophy regions in right MTLE-HS (r-MTLE-HS) subjects (*p* < 0.005)	

vs. Ctrls	vs. left MTLE-HS (l-MTLE-HS) subjects
Right hippocampus [26, −28, −14]	Right hippocampus [28, −26, 14]
Right-inferior temporal gyrus [54, −44, −14]	Left central sulcus [−42, 20, 12]
Cerebellum bilaterally [−18, −64, −44] [24, −62, −44]	Left precentral gyrus [−44, 32, 22]

**Relative atrophy regions in l-MTLE-HS subjects (***p*** < 0.005)**	

**vs. Ctrls**	**vs. r-MTLE-HS subjects**

Left hippocampus [−32, −26, −14]	Left-inferior temporal gyrus [−54, −28, −18]
Right-inferior temporal gyrus [5, −48, −10]	Left insula [−40, 16, 10]
Right corpus callosum [8, −10, 28]	Left putamen [−20, 8, −8]
Insula bilaterally [−34, −8, 10] [28, −8, 10]	
Cerebellum bilaterally [−28, −60, −44] [26, −60, −44]	

**Figure 2:**
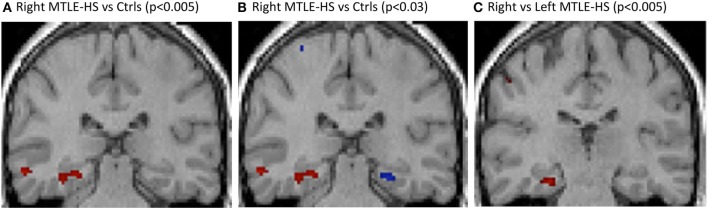
VBM T1-weighted multiplanar images comparing **(A)** r-MTLE-HS patients vs. healthy controls, showing relative right hippocampal atrophy; **(B)** r-MTLE-HS patients vs. healthy controls with *p* < 0.005 in red and *p* < 0.03 in blue, showing bilateral relative hippocampal atrophy; **(C)** r-MTLE-HS vs. l-MTLE-HS patients, showing relative right hippocampal atrophy. SPM results are superimposed on a T1 3d image of one of the healthy subjects. l-MTLE-HS, left MTLE-HS patients; r-MTLE-HS, right MTLE-HS patients; SPM, statistical parametric map; VBM, voxel-based morphometry.

Right MTLE-HS patients showed right hippocampal atrophy (Figure 2A) as compared with healthy controls, and when the statistical significance threshold was lowered to *p* < 0.03, contralateral hippocampal atrophy was also detected (Figure 2B). r-MTLE-HS patients showed relative right hippocampal atrophy as compared with l-MTLE-HS patients (Figure 2C).

Regions with significantly reduced gray-matter volume involved several extrahippocampal brain regions in addition to the ipsilateral hippocampus.

Compared with healthy controls, r-MTLE-HS patients had regions with significantly reduced gray-matter volume also in the right-inferior temporal gyrus, and in the ipsi- and contralateral cerebellum. Compared with l-MTLE-HS patients, they showed reduced gray-matter volume of the left central sulcus and the left precentral gyrus.

Left MTLE-HS patients showed relative left hippocampal atrophy as compared with healthy controls and not compared with r-MTLE-HS patients. When the statistical significance threshold was lowered to *p* < 0.03, no contralateral hippocampal atrophy was detected.

Compared with healthy controls, l-MTLE-HS patients had regions with significantly reduced gray-matter volume also in the left-inferior temporal gyrus and corpus callosus and in the ipsi- and contralateral insula and cerebellum. Compared with r-MTLE-HS patients, they showed reduced gray mater volume of the left-inferior temporal gyrus, insula, and putamen.

Correlation analysis in MTLE-HS subjects showed no significant effects. In particular, no significant correlation was found between ipsilateral H and PH gray-matter volume and 30 MGS ED, number of anti-epileptic drugs, seizure frequency, age, age at epilepsy onset, and disease duration.

### MGS ED and VBM Data

The PHC volumes were invariably larger in the right than in the left hemisphere both in controls and in patients (Table 5).

**Table 5:** Mean and SE values of the right and left volumes and volume difference between right and left parahippocampus (PH) in controls (Ctrl), right (r MTLE-HS), and left (l MTLE-HS) MTLE-HS patients.

	Group	Mean	SE
Right PH volume	Ctrl	650.380	2.230
	r-MTLE-HS	621.000	4.170
	l-MTLE-HS	655.330	3.160

Left PH volume	Ctrl	570.850	1,600
	r-MTLE-HS	580.430	3.500
	l-MTLE-HS	551.000	3.340

Right–left PH volume difference	Ctrl	40.570	2.090
	r-MTLE-HS	104.330	3.040
	l-MTLE-HS	76.530	1.830

The right and the left PHC volumes were not different in patients with and in those without abnormal 30 MGS ED values (Table 6). By contrast, the patients with abnormal 30 MGS ED values showed a right–left PHC volume difference smaller than those with normal 30 MGS ED, namely abnormal 30 MGS ED values are associated with smaller right PHC volumes as expected on the basis of their left PHC volume. All the four patients with abnormal 30 MGS ED belonged to the r-MTLE-HS group, and three of them showed the smallest volume difference, whereas the other one showed the largest difference and the shortest disease duration.

**Table 6:** Mean and SE values of right and left volume and volume difference between right and left parahippocampus (PH) in MTLE-HS patients showing impaired 30 MGS ED and in MTLE-HS patients not showing impaired 30 MGS ED.

	Impaired ED in 30 MGS	*N*	Mean	SE	*t*	*p*
Right PH volume	No	9	646.560	7.370	1.596	0.186
	Yes	4	615.000	18.340		

Left PH volume	No	9	563.670	9.010	0.628	0.554
	Yes	4	574.000	13.770		

Right–left PH volume difference	No	9	82.889	11.760	2.667	0.024
	Yes	4	41.000	10.320		

Finally, concerning the hippocampus, none of the volume parameters, including the right–left difference, proved to be different depending on the abnormality of 30 MGS ED.

## Discussion

Memory-guided saccades abnormalities together with VBM results in our study (i) suggested the functional involvement of the right PHC in patients with right MTLE-HS, (ii) supported a right lateralization of spatial memory control, and (iii) showed a relation between functional impairment and degree of atrophy.

Our results showed in detail that the saccade velocity and latency values in MTLE-HS patients were not different from controls for all kind of saccades, thus suggesting that the cortical and brainstem mechanisms to program and trigger saccades were not affected.

Moreover, MTLE-HS patients do not differ from controls for the accuracy of RS and of 3 MGS: no differences could be found for the accuracy of the first saccade (pulseE), and for the improvement that could be obtained after corrective saccades (finalE), and the capability to improve the accuracy of the first saccade (ED). The finalE of RS showed a larger accuracy for rightward than for leftward saccades, but this difference was the same in patients and in controls.

Interestingly, r-MTLE-HS patients differ from controls for the accuracy of 30 MGS. Both the patients and the controls showed a pulseE and a finalE that were smaller for leftward than for rightward saccades; that is leftward 30 MGS were more accurate both after the first and after all the corrective saccades were made; however, the r-MTLE-HS patients were less effective in improving the accuracy of the first saccade than both the controls and the l-MTLE-HS patients. This finding is supported by ED value evaluation both in the group and in the individual analyses. Concerning the group analyses, it is noteworthy that, even if it proved to be statistically significant only for the leftward direction, the mean ED values of 30 MGS from r-MTLE-HS showed a similar trend for the rightward direction also.

The individual values showed that four out of seven r-MTLE-HS patients (vs. none of l-MTLE-HS patients and controls) had an abnormally positive ED value, meaning that in these subjects the corrective saccades did not improve and even worsen the accuracy of the position reached by the first saccade. In all of these four patients, ED was abnormal for l–30 MGS; in one of them the abnormality was bilateral, and this specific r-MTLE-HS patient was the one showing the smallest PH volumes not only for the right side but also for the left side.

Memory-guided saccades results suggest the functional involvement of the right PHC in patients with r-MTLE-HS, in keeping with the results of a previous study by our group (20) and, in agreement with previous observations (19, 23), our data suggest a possible specialization of the right PHC for visual spatial memory (24–29).

Voxel-based morphometry analysis results confirmed the presence of hippocampal atrophy as detected by conventional MRI in our patients and were in accordance with the results of previous imaging studies. Furthermore, VBM analysis showed that, despite no PHC alteration detected by conventional MRI, the mean volume of the parahippocampus was smaller in patients with impaired accuracy of 30 MGS.

Many neuroimaging studies showed that MTLE-HS patients have also areas of extrahippocampal atrophy, including the PHC (30–36). The presence of extrahippocampal structural damage in MTLE-HS has been correlated with postsurgery outcome in these patients; for instance, it was demonstrated that seizures after surgery commonly arise within the spared structures of the resected temporal lobe (37, 38) and patients with extrahippocampal atrophy had a lower probability of becoming seizure free after complete hippocampal resection (39).

Thereby, MGS and VBM could be useful in presurgical evaluations aimed at deciding the extension to extrahippocampal structures of the surgical resection.

Temporal lobe atrophy is considered the result of an apoptotic mechanism due to frequent seizure recurrence (40–43) and FS are considered as a precipitating insult for the neuronal loss in MTLE-HS patients (44). Since hippocampal atrophy in MTLE-HS patients is associated with white matter fiber disconnections, an alternative hypothesis is that deafferentation from hippocampal fibers could be the major determinant of extrahippocampal atrophy (45). Clinical variables such as FS, age at seizure onset, disease duration, and seizure frequency were not correlated with MGS and VBM parameters in our patients, but these results could be biased by a relatively small sample size in our study. Repeated evaluations in MTLE-HS patients could give some information regarding the evolution over time of the cortical functional and structural damage.

## Ethics Statement

The research protocol received approval from the local Ethics Committee and all the procedures were conducted in accordance with the Declaration of Helsinki. All the subjects gave written informed consent before participating in the study.

## Author Contributions

SC and MV contributed to the conception or design of the work and made acquisition, analysis, and interpretation of data for the work. GB, AP, GP, SB, and CAG contributed to the acquisition, analysis, and interpretation of data for the work. SC, GB, AP, GP, SB, CAG, and MV drafted the manuscript, critically reviewed the manuscript for important intellectual content, approved the final version of the manuscript, and agreed to be accountable to all aspects of this work ensuring that questions related to the accuracy or integrity of any part of the work are appropriately investigated and resolved.

## Conflict of Interest Statement

The authors declare that the research was conducted in the absence of any commercial or financial relationships that could be construed as a potential conflict of interest.
